# Spatiotemporal modeling of urban green space connectivity and landscape structure for climate adaptation in Tehran, Iran

**DOI:** 10.1371/journal.pone.0341276

**Published:** 2026-04-09

**Authors:** Zahra Omidighalehmohammadi, Maryam Morovati, Peyman Karami, Zahra Parvar

**Affiliations:** 1 Department of Environmental Sciences and Engineering, Faculty of Agriculture and Natural Resources, Ardakan University, Ardakan, Iran; 2 Department of Environmental Sciences, Faculty of Natural Resources and Environment Sciences, Malayer University, Malayer, Iran; 3 Department of Environmental Sciences, Faculty of Fisheries and Environmental Sciences, Gorgan University of Agricultural Sciences and Natural Resources, Gorgan, Golestan, Iran; Sejong University, KOREA, REPUBLIC OF

## Abstract

Urban heat island (UHI) effects, exacerbated by climate change, highlight the urgent need for effective management of urban green spaces (UGSs) to regulate city temperatures. However, previous studies primarily concentrated on the effects of landscape composition and configuration on mitigating urban heat islands. To address this gap, this study develops a connectivity-based framework to examine the spatiotemporal evolution of urban green spaces in Tehran, Iran, using satellite data from 2015 to 2023 processed in Google Earth Engine. Landsat-derived land surface temperature (LST) and NDVI maps were used to identify urban heat and cool islands. ROC and TSS metrics were applied to determine an optimal NDVI threshold for classifying green and non-green areas. Landscape morphology metrics quantified spatial configuration changes, while Foreground Area Density and inverse NDVI maps were used to detect core green spaces and resistance surfaces, respectively. Connectivity flows between cold cores were modeled using electrical circuit theory. Results revealed a significant decrease of 1,400 ha in cold areas in northern Tehran and a 1,618 ha increase in hot zones in the south and southwest. Spatial analysis revealed increased cold patch fragmentation and hot zone expansion, reducing green space continuity and intensifying the UHI effect. While cold cores in 2015 showed weak, scattered connectivity, by 2023 they became more concentrated in northern Tehran with a marked increase in current flow, indicating improved local connectivity. However, this concentration reveals growing imbalance in UGS distribution and cooling capacity, highlighting the need for targeted green infrastructure in southern, western, and central areas. The findings offer valuable insights for urban planners to enhance UGS connectivity and mitigate UHI effects, supporting climate resilience and improving urban quality of life.

## 1. Introduction

Urbanization, driven by population growth and the demand for improved living standards, has led to profound changes in city size, density, and land use [1]. The conversion of natural landscapes, including agricultural lands and forests, into urban areas has introduced significant environmental challenges [2].Climate change further compounds these issues by altering ecosystems and local climates, affecting both air temperatures and biodiversity [3]. A comprehensive understanding of urban climate dynamics is essential for mitigating these impacts and improving the quality of life in rapidly growing metropolitan areas [2,4]. Urban Heat Island (UHI) intensification—driven by rapid urbanization and population growth—has become a pressing concern, with cities exhibiting significantly higher temperatures than their rural surroundings [5–7]. While traditional meteorological data offer high temporal resolution, their limited spatial coverage has prompted a shift toward satellite-based Land Surface Temperature (LST) measurements, which provide broader spatial insights into urban thermal patterns [7,8]. LST, reflecting surface-atmosphere energy exchanges, has thus emerged as a critical metric for UHI assessment in urban climate studies [2,9,10].

One of the most critical consequences of UHIs is the increase in heat stress and air pollution in urban areas [11]. To counteract these effects, enhancing urban cool islands—such as parks, gardens, rivers, and other vegetated or water-covered areas—has proven to be a practical and effective strategy [12,13]. The cooling performance of Urban Green Spaces (UGSs) largely depends on vegetation structure, species composition, and spatial configuration. Therefore, precise assessment of their cooling capacity is essential for effective urban planning and climate adaptation [14–16].

Studies have shown that the cooling service function of UGSs is highly dependent on their spatial patterns within the urban landscape [17–20]. In rapidly urbanizing regions, changes in landscape structure and function have emerged as key drivers of rising LST [21–23]. These shifts underscore the need to explore the dynamic spatial and temporal relationships between landscape patterns and LST, offering valuable insights into how specific landscape metrics contribute to urban thermal regulation [24].

Landscape composition and configuration are key factors in assessing the impact of LST. While composition refers to the proportion of different land cover types, configuration involves the shape, density, and spatial heterogeneity of landscape elements [25]. Expanding UGSs can significantly reduce LST, and optimizing the geometric design of green and blue infrastructures can enhance their cooling performance [24]. Previous studies have examined landscape metrics affecting LST in both urban and rural settings and proposed various strategies to mitigate urban thermal conditions through landscape planning [17,24–33].

Rao et al. (2021) examined the influence of urban growth patterns on LST in 338 Chinese cities and found that compact, edge, and infill developments generally increased surface temperatures. The impact of scattered growth varied across climate zones, with negative effects in temperate areas and positive in arid regions. Their study also emphasized the significance of parcel-level analysis for more accurate assessments and urban planning [31].

Numerous studies in cities such as Nanchang, China [21], Shiraz, Iran [17], Harbin, China [34], and Constantine, Algeria [35] have confirmed that vegetated areas are markedly more effective in lowering LST than built-up or barren surfaces. However, the thermal efficiency of green spaces is not solely determined by their extent but also by their spatial structure. Evidence from recent literature [17,25,28,29,36] highlights the crucial role of landscape metrics such as patch size, connectivity, adjacency, and vegetation composition in shaping urban thermal patterns. Particularly, connected and structurally coherent green patches tend to exert more consistent and widespread cooling effects.

Moreover recent studies in Iranian cities have highlighted the increasing significance of UHIs under rapid urbanization [1,37,38]. In Isfahan, Shirani-Bidabadi et al. (2019) examined the expansion of heat-affected areas in sparsely vegetated zones [39]. Bokaie et al. (2016) emphasized that Tehran’s polycentric and topographically diverse structure, combined with limited UGSs, high population density, emissions, and extensive impervious surfaces, contributes to heightened UHI vulnerability [9]. Parvar et al. (2025) in Bojnourd analyzed the role of spatial configuration and connectivity of green patches in regulating local temperatures [22]. Similarly, studies in Karaj [18] and southeastern Iran emphasized [40] have investigated the influence of green space arrangement, patch connectivity, and landscape metrics on urban thermal patterns [18,72]. These investigations collectively underscore the growing research attention to UHIs and the role of urban green infrastructure in Iranian urban contexts.

Despite these insights, a significant gap remains in understanding the spatiotemporal dynamics of green space connectivity and its direct influence on UHI mitigation. Most existing studies adopt static or localized analyses and often overlook the evolving configuration of green networks and their systemic role in urban temperature regulation. For example, Mokhtari et al. (2022) investigated the role of green spaces in Karaj, Iran, yet did not explore broader connectivity frameworks or long-term dynamics [18].

Addressing this gap, the present study offers a novel integration of circuit theory with spatial landscape analysis to model UGSs connectivity in Tehran over time.

Circuit-based approaches—though rarely applied in urban thermal studies—provide a more realistic simulation because they consider multiple potential pathways of heat diffusion rather than a single least-cost route [41]. By modeling thermal flow as electrical current spreading through a resistance surface, this method captures the complex and multidirectional nature of heat transfer in heterogeneous urban landscapes. At the same time, it highlights the importance of connectivity: well-connected green patches enable cooling effects to spread effectively, while fragmented ones remain isolated with limited impact [40]. Thus, analyzing connectivity not only helps identify priority areas for strengthening green heat sink networks, but also advances scientific understanding of how spatial configuration influences ecological functions in urban systems [18].

Using multi-temporal satellite data and advanced geospatial techniques within Google Earth Engine (GEE), this study develops a robust framework for identifying spatial priorities for green space development. The research is guided by three central questions: 1-What are the spatiotemporal patterns of UHI in the megacity of Tehran? 2- How do landscape metrics help explain urban thermal environments? 3- How has the cooling potential of cold patch connectivity evolved over time? By bridging theoretical innovation with practical urban challenges, this study contributes a replicable, evidence-based model to inform climate-resilient planning and optimize UGSs networks for sustainable thermal regulation.

## 2. Materials and methods

### 2.1. Study area

**Fig 1:**
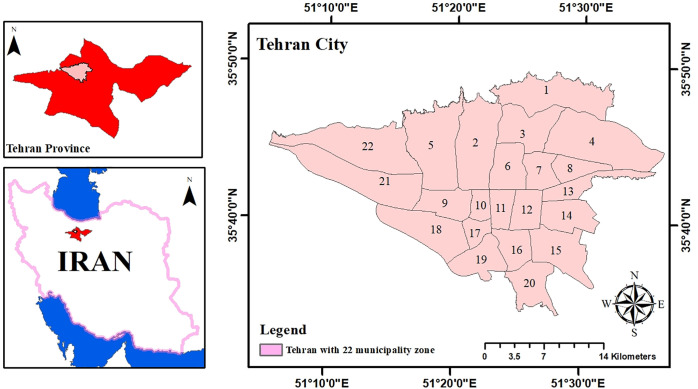
Geographic location of Tehran city within Iran and Tehran province (Data source: (Iran administrative boundaries, Tehran city basemap, Tehran municipal districts shapefile).

Tehran, Iran’s capital and largest metropolis (~730 km^2^), is home to approximately 8.69 million residents, distributed across 22 districts and 375 neighborhoods. The city exhibits a significant population density, averaging 119 persons per hectare, positioning Tehran among densely populated metropolises by global standards [42]. Its topography, ranging from 1,050–1,800 meters between the Alborz Mountains in the north and the central desert plateau in the south, creates pronounced thermal contrasts, with cooler, more humid conditions in northern districts and hotter, drier conditions in southern areas. As of 2021, Tehran contained around 6,017.5 hectares of parks and urban green spaces, translating to an average per capita green space of 6.93 m^2^well below the 10 m^2^ target defined by the city’s Master Plan. The city’s green infrastructure includes public parks, neighborhood gardens, and urban forests, providing recreational and ecological services across Tehran’s districts [43]. Rapid urban expansion and fragmentation of green spaces, combined with this semi-arid to Mediterranean climate, contribute to intensified Urban Heat Island effects, making Tehran an ideal case for analyzing how urban green space connectivity and landscape structure influence thermal regulation over time (Fig 1).

### 2.2. Methodology

This study used multi-temporal Landsat-8 OLI and TIRS imagery to analyze LST and vegetation changes in Tehran during the summers of 2015 and 2023. Cloud-free Level-1 images (≤10% cloud) with 30 m resolution were processed on Google Earth Engine (GEE). Preprocessing included mosaicking and radiometric calibration.

LST was retrieved using TIRS Band 10 via the mono-window algorithm, incorporating surface emissivity estimated from NDVI. NDVI, derived from Bands 4 and 5, was used to assess vegetation health and inform emissivity and green core extraction.

**Fig 2:**
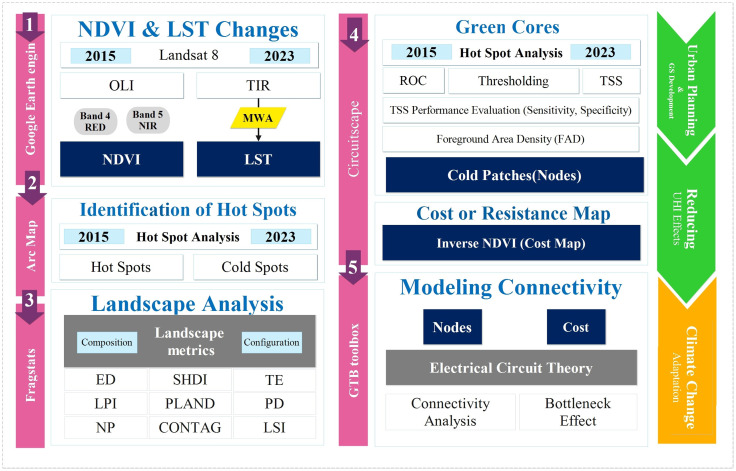
Flowchart of the different stages of implementing the method of this study. (Created entirely by the authors using Microsoft Office tools).

The methodological workflow, as illustrated in Fig 2, involved 1) Generating LST and NDVI maps, 2) identifying UHIs via Getis-Ord Gi* spatial statistics, 3) extracting high-NDVI green cores, 4) modeling connectivity with Circuitscape, using resistance surfaces from NDVI, land cover, and LST and 5) quantifying landscape change and fragmentation with FRAGSTATS and ArcGIS.

The overall methodological workflow is illustrated in Fig 2, summarizing the sequential steps from data acquisition and preprocessing to landscape metrics analysis and connectivity modeling.

#### 2.2.1. Retrieving LST and preparing NDVI.

For LST retrieval, a Mono-window algorithm (MWA) was implemented, which utilizes Band 10 of the TIRS and does not require atmospheric profile parameters, making it efficient for large-scale applications [8,44] (see Equation 1):


$${LST\;(T}_{s})=BT/(\text{1}+\lambda \times \left(\frac{BT}{\rho }\right)\times ln\left(\varepsilon_{\lambda }\right))$$ (1)

Where:

Ts is the LST;

*BT* represents the brightness temperature (in Kelvin);

λ denotes the wavelength of the emitted radiance.

The coefficient (*ρ*) is equal to ($\text{1}.\text{438}\times {\text{10}}^{-\text{2}}mk$)

$\varepsilon_{\lambda }\;$is the emissivity.

NDVI, calculated from Landsat 8 Bands 4 and 5, quantifies vegetation density (range: −1 to +1) [45,46]. Higher values indicate healthier vegetation; negatives often represent water [37–40]. NDVI maps for 2015 and 2023 were derived using Equation (2) [47,48].


$$NDVI=\frac{NIR-RED}{NIR+RED}$$ (2)

Vegetation cover plays a crucial role in regulating LST as denser vegetation generally corresponds to lower LST values due to increased evapotranspiration and surface shading [49].

#### 2.2.2. Identification of hot and cold spots.

After extracting the LST maps for the study area, the identification of hot and cold spots was conducted to investigate the spatial distribution and connectivity of green, thermally cool patches. A fishnet grid was created in ArcMap by generating points at the centroid of each LST pixel, resulting in a uniform point layer containing temperature values. This point dataset was used as input for Hot Spot Analysis using the Getis-Ord Gi* statistic. The Gi* values were calculated at three significance levels (90%, 95%, and 99%) to detect statistically significant hot (high-temperature clusters) and cold (low-temperature clusters) spots [50]. Clusters with high positive Gi* values were identified as hot spots, while those with low negative values were identified as cold spots.

In this study, only cold spots with a confidence level of 95% and higher (Gi* = –2 and –3) were selected for further spatial analysis and subsequent steps.

After identifying cold and hot thermal islands for monitoring and analyzing their spatial changes, these maps were converted into raster layers, and their overlap was then calculated using the Kappa coefficient.

#### 2.2.3. Analysis of the landscape patterns.

To assess the spatial configuration and dynamics of heat and cool island patterns, hotspot maps from 2015 and 2023 were analyzed using landscape metrics in Fragstats software. Landscape metrics quantify the composition and configuration of spatial elements, offering insights into landscape heterogeneity [51]. Given the scale dependency and potential redundancy among some metrics, nine widely-used indices were selected at both class and landscape levels, based on previous studies [25,28,30]. Table 1 summarizes the selected landscape metrics.

**Table 1:** Details of landscape metrics used in the study.

Metric Name	Abbreviation	Unit	Range	Concept
Number of patches	NP	None	Greater than zero	Fragmentation
Largest patch index	LPI	Percent	0–100	Dominant cover or land use; patchiness
Patch density	PD	Number per 100 hectares	Greater than zero	Patchiness of the landscape
Shannon’s diversity index	SHDI	None	Greater than or equal to zero	Landscape diversity
Landscape shape index	LSI	None	Greater than or equal to one	Patchiness and shape of the landscape
Contagion	CONTAG	None	0–100	Patchiness and connectivity of the landscape
PLAND	PLAND	Percent	0–100	Relative abundance of each class in the overall landscape
Total Edge	TE	Meter	Greater than or equal to zero	Landscape configuration
Edge Density	ED	Meter	Greater than or equal to zero	Amount of edge

#### 2.2.4. Identification of green cold cores (GCCs).

Green cool spots identified at the 95% confidence level and above in the previous step were used as the basis for this stage. To identify and delineate UGSs, 250 training samples of green areas (e.g., parks, boulevards, gardens, and vegetation) and 250 samples of non-green areas (e.g., bare soil, buildings, and asphalt) were labeled using Landsat false color composites and Google Earth imagery. These samples formed a Boolean layer and were integrated with LST and NDVI maps to discriminate between green and non-green areas. A thresholding method based on Mokhtari et al. (2022) was applied to determine the precise boundary between the two classes [18]. The optimal threshold was identified using Receiver Operating Characteristic (ROC) curve analysis and the True Skill Statistic (TSS). The ROC curve evaluates classification performance by illustrating the trade-off between sensitivity and false positives, while TSS—calculated from the confusion matrix—assesses classification accuracy by computing sensitivity, specificity, and overall accuracy [52]. TSS was calculated as shown in Equation (3).


TSS=Sensitivity+ Specificity−1 (3)

Since the confusion matrix is influenced by the threshold value, the optimal threshold for calculating TSS must be determined. Typically, the threshold that maximizes the sum of sensitivity and specificity is recommended [52].

The calculation of sensitivity and specificity is based on the following: (a) True Positive (TP): the model predicts green space presence, and the test data confirms it. (b) False Positive (FP): the model predicts green space presence, but the test data shows absence. (c) False Negative (FN): the model predicts absence, but the test data shows presence. (d) True Negative (TN): the model and test data both predict absence. Evaluation metrics are derived from these variables (a, b, c, and d).

Sensitivity (H) refers to the proportion of correctly identified green areas [52], calculated as shown in Equation (4):


$$H\;=\frac{a}{a+c}$$ (4)

False Positive Rate (F) refers to the proportion of non-green areas misclassified as green [52], calculated as shown in Equation (5):


$$\text{F }=\frac{\text{b}}{\text{b}+\text{d}}$$ (5)

The TSS, similar to the Kappa coefficient, accounts for both omission and commission errors and ranges from −1 to +1. A TSS of +1 indicates perfect agreement, while values of 0 or below suggest performance no better than random. TSS values below 0.2 indicate poor performance, values between 0.2 and 0.6 reflect moderate performance, and values above 0.6 indicate strong model performance [53].

Based on the sensitivity and specificity values, the NDVI map demonstrated superior performance in distinguishing green from non-green areas compared to the LST map and was therefore selected for further analysis. Following the identification of green cold cores (GCC), the Fragmentation and Aggregation Degree (FAD) analysis was applied using GTB Toolbox 3.3 to reduce patch numbers and emphasize dominant ones [54].

This method employs moving windows of five neighborhood sizes (7, 13, 27, 81, and 243 pixels) to capture spatial heterogeneity at multiple scales and classifies patches into six density categories: rare, patch, transitional, dominant, interior, and intact [55]. Retaining only the more aggregated categories (transitional, dominant, interior, and intact) ensures that small and scattered patches, which contribute minimally to cooling and connectivity, are excluded, while larger, cohesive green cores are preserved. These core patches serve as the nodes for subsequent connectivity analysis, allowing the construction of green networks that effectively represent potential pathways for cooling flow across the urban landscape [41,56]. In ArcGIS Pro, reclassification tools were then used to isolate the main core patches with a dominance degree of 60 or higher for further steps.

#### 2.2.5. Modeling the connectivity of GCCs using electrical circuit theory.

The connectivity of UGSs is a fundamental aspect of landscape pattern analysis and plays a significant role in regulating LST [57]. In this study, circuit theory was applied using Circuitscape software to analyze the connections among significant GCCs and to identify optimal connectivity pathways. By modeling current flow between landscape nodes, the degree of connectivity and its influence on LST were evaluated [58].

Step 1. Converting the Landscape into an Electrical Graph: In this step, each pixel in the raster map prepared in the previous phase was considered as a node. Then, adjacent pixels were connected to form an electrical network, which enabled the analysis of current flow between nodes (Malkooti-Khah et al., 1392).

Step 2. Defining the Cost or Resistance Map of the Landscape: The cost or resistance resistance map reflects the difficulty of cooling flow between GCCs, incorporating both voltage (cooling potential) and resistance (landscape permeability) [18,59]. Due to the inverse NDVI–LST relationship, resistance was modeled using the inverse NDVI values, where higher resistance corresponds to sparsely vegetated areas [60–62].

Step 3. Modeling Connectivity and Cooling FlowL: In circuit theory, four main modes—Pairwise, One-to-all, All-to-one, and Advanced—can be used to calculate connectivity between nodes [41,63]. In this study, the Pairwise Mode in Circuitscape was applied to simulate current flow between each pair of main GCC nodes. This approach provides a detailed, pair-by-pair assessment of connectivity, allowing the identification of optimal pathways where cooling effects are most effectively transmitted. Areas with the highest current density were designated as green corridors, while narrow pathways with concentrated flow highlighted bottlenecks that reduce landscape connectivity and limit cooling efficiency. Using this method, critical zones were identified where targeted interventions—such as vegetation enhancement—would most effectively improve the connectivity of green patches and strengthen the overall cooling capacity of the urban landscape [41,63].

## 3. Results

### 3.1. Dynamics of vegetation and UHIs

**Fig 3:**
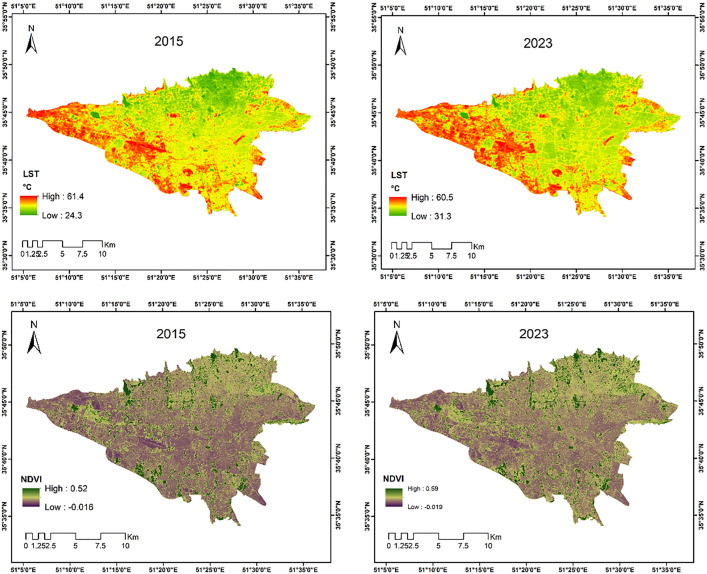
NDVI and mean LST maps in the summer quarter of 2015 and 2023. (Derived from publicly available Landsat 8 imagery accessed and processed on GEE; original data by USGS/NASA.).

Fig 3 shows the NDVI maps and the mean LST for the summer season (three-month period) in 2015 and 2023. In 2015, the LST values ranged between 24.3°C and 61.4°C, while in 2023 these values ranged between 31.3°C and 60.5°C. A significant difference in temperature between the northern and northeastern parts of the Tehran metropolitan area and its western regions is evident. Specifically, in both 2015 and 2023, the air temperatures in the northern, northeastern, and parts of central Tehran were noticeably lower than in other areas, whereas the temperatures in the southern and southwestern parts were considerably higher. The NDVI map illustrates the heterogeneity of vegetation cover in the study area. In 2015, the minimum and maximum NDVI values were 0.16 and 0.52, respectively, and in 2023, they were 0.19 and 0.59.

**Fig 4:**
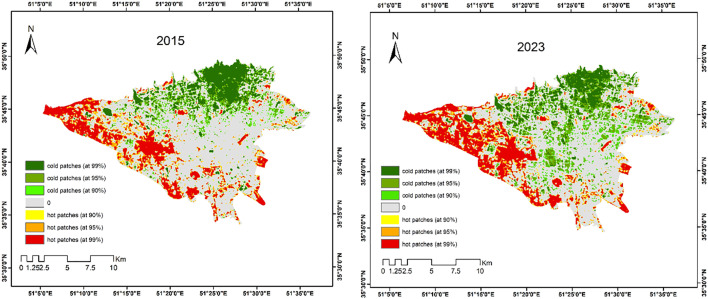
Cooling and Heat Islands in Tehran (2015 & 2023). (Map Created by the authors using Landsat 8 LST data accessed via GEE).

A feature with a high value is not considered a hotspot unless both the feature and its neighboring features are statistically significant. Fig 4 shows hot and cold spots at three significance levels for 2015 and 2023. In 2015, urban heat islands were mainly concentrated in the southern, southwestern, and western parts of the city. This pattern remained in 2023, with increased extent and connectivity of hot spots. Additionally, cold areas expanded in 2023, including into parts of central Tehran, across varying levels of significance.

As shown in the Fig 4, cold spots are mostly concentrated in the northern areas, while hot spots are clustered in the southwestern parts of Tehran. The areas of hot and cold spots at different confidence levels were calculated and are presented in Table 2. Results for 2015 and 2023 indicate a decline in green (cool) areas at the 99% confidence level, alongside an expansion of urban heat islands.

**Table 2:** Area of hot and cold spots at varying significance levels (2015 and 2023).

Significance Level (Z-score)	Area (ha) – 2015	Area (ha) – 2023
−3	6988.93	5588.12
−2	3789.06	5800.74
−1	2578.60	4336.27
0	35597.91	31597.83
1	1989.25	1733.29
2	3688.80	3453.80
3	9058.24	10676.92

**Fig 5:**
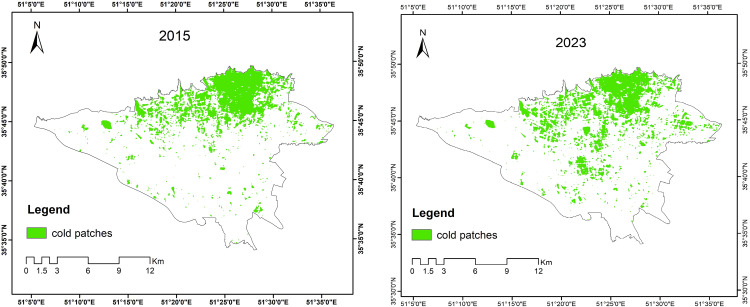
Cold spots (≥95% confidence), 2015 vs. 2023. (Generated by the authors using the Getis-Ord Gi* statistic on the LST map from Landsat images (USGS)).

Fig 5 illustrates the spatial distribution of green patches with ≥95% confidence considered in the analysis. While the area of cold spots expanded from 2015 to 2023, their connectivity in northern Tehran declined, leading to fragmentation. Conversely, green space connectivity in the city center improved.

Table 3 presents the results of the Kappa coefficient, which measures the spatial overlap between the cold spot maps for 2015 and 2023. The Kappa value of 0.84 indicates substantial agreement between green and non-green spaces, suggesting that UGSs have undergone limited spatial changes over the two years.

**Table 3:** Kappa coefficients of changes in cold spaces.

		2023		Total
		Non-GS	GS	
**2015**	Non- GS	0.92	0.01	0.94
	GS	0	0.05	0.05
**Total**		0.92	0.07	1
**Overall Kappa**				0.84

### 3.2. Identification of GCCs (connectivity nodes)

**Fig 6:**
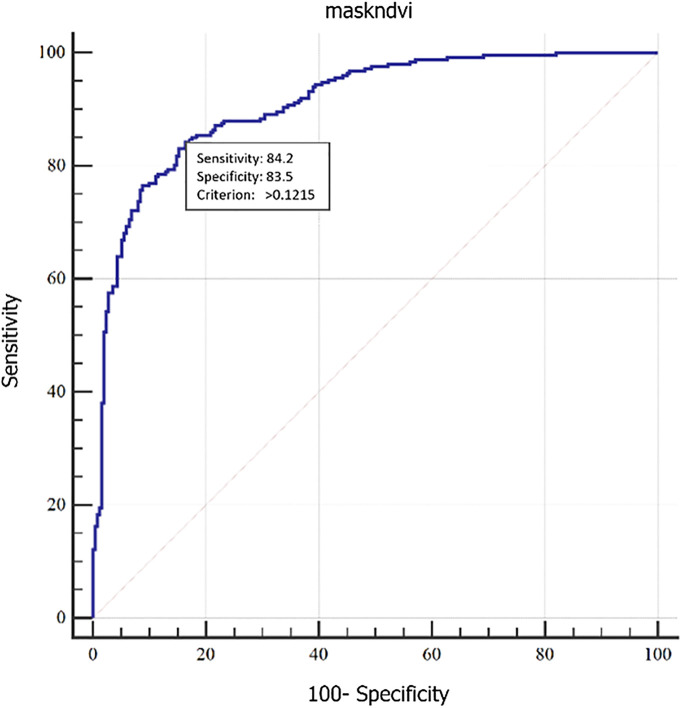
Diagnostic test of green and non-green space using TSS threshold in 2015-2023.

Fig 6 displays the threshold used to separate green from non-green areas for the years 2015 and 2023. In this figure, the cutoff point—where the sensitivity and specificity values are at their maximum—is shown. Specifically, this cutoff indicates that in 2015, the NDVI index for values above 0.1238 could differentiate green spaces with a sensitivity of 81.2% and distinguish non-green areas with a specificity of 83.9%. Likewise, in 2023, the NDVI index for values above 0.12 distinguishes green spaces with a sensitivity of 84.2% and non-green areas with a specificity of 83.5%.

Table 4 displays the test statistics for this evaluation.

**Table 4:** TSS Threshold results for green spaces in 2015–2023.

Criteria	2015	2023
AUC	0.89	0.91
TSS	0.85	0.87
Threshold	>0.1238	>0.1215
Sensitivity	% 81.2	% 84.2
Specificity	% 83.9	% 83.5

After applying the threshold values, a final map of green patches was created. The maps reveal that green spaces are predominantly concentrated in the northern parts of the city, with the southern areas showing a deficiency in both 2015 and 2023. The comparison between the two years indicates a reduction in the size and area of UGSs in the southern part of the city by 2023.

**Fig 7:**
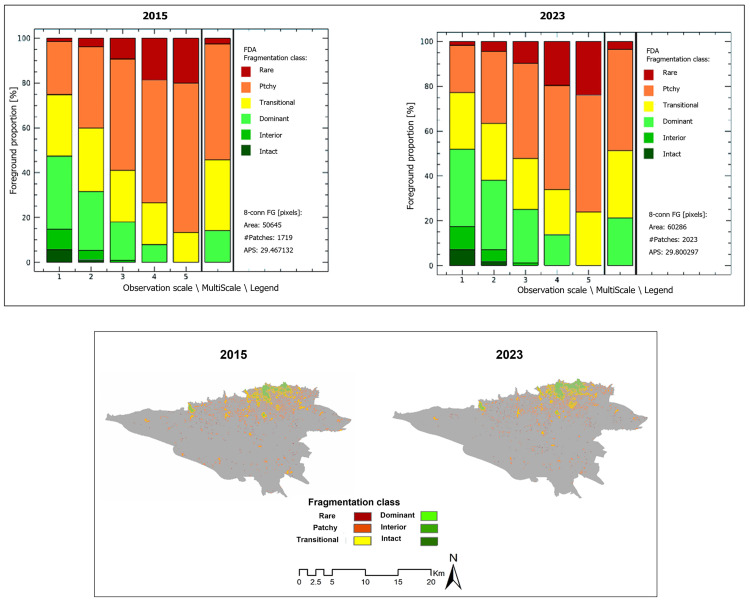
Multi-scale analysis (FAD) of cold patches for 2015 and 2023. (Created by the authors using GuidosToolbox^‌‌^ (publicly available).

Due to the high number of identified green patches in the previous step, a multi-scale analysis of cold patches was performed for ease and to enable further analysis. Fig 7 presents the results of the multi-scale analysis (FAD) of cold patches for 2015 and 2023. The maps and chart show values within class intervals ranging from 0 to 100, with intervals of 0–20, 20–40, 40–60, 60–80, and 80–100 representing different densities of cold green patches. As shown, cold green spaces in the southern part of the city are fragmented and sparse, while in the northern parts, high-density patches are concentrated.

Fifteen GCCs were identified based on the FAD analysis for both years. Ultimately, the primary cores, characterized by large and extensive patches with high dominance (above 60), were selected for further analysis.

### 3.3. Modeling connectivity

The highest cost was 0.5 in 2015 and 1.04 in 2023, indicating a decrease in vegetation cover. Higher costs reflect lower vegetation density, leading to reduced cooling effects and greater difficulty in connecting cool cores. The cost in 2023 was nearly double that of 2015, suggesting a significant decline in the cooling function of green spaces in Tehran over the 8-year period.

**Fig 8:**
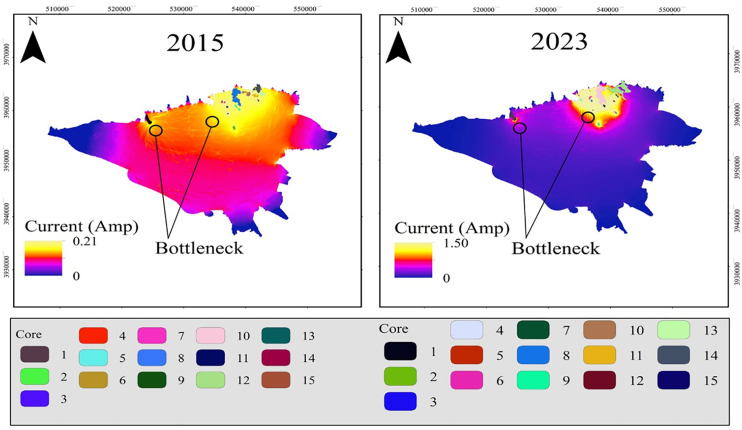
Electrical circuit theory maps for 2015 and 2023. (Map generated by the authors using the open-source Circuitscape software and shapefiles from OpenStreetMap (OSM)).

Fig 8 illustrates the results of applying circuit theory to model connectivity between cool nodes using the cost maps and green cool patches as core nodes for 2015 and 2023.

The final output of the circuit theory model was a current density map (in amperes) for each year, where higher currents indicate stronger connectivity. Bottlenecks, where flow must pass through narrow green structures, were also identified—these typically correspond to GCCs visible in hotspot maps. As shown in Fig 8, connectivity between GCCs was significantly lower in 2015, with scattered green spaces across western and central Tehran and a maximum current of 0.2 A. In contrast, by 2023, current intensity increased to 1.5 A, with flows concentrated in the northern part of the city. This shift highlights a reduction in green space distribution and a move toward concentration in the north, reflecting changes in urban development patterns. While this may improve localized connectivity, it also suggests spatial imbalance in green space distribution. Bottlenecks were more scattered in 2015 but became less pronounced in 2023 due to altered flow paths.

### 3.4. Analysis of changes in the landscape patterns of thermal and cooling Islands

Landscape metrics were analyzed at both class and landscape levels using hotspot areas with 95% and 99% confidence for the years 2015 and 2023. Table 5 presents the results of landscape metrics analysis at these significance levels.

**Table 5:** Class-level Landscape Metrics Values.

Class Metric	year	Cold & Hot spot level			
		Cold spot		Hot spot	
		−3 (%95)	−2 (%95)	2 (%95)	3 (%95)
PLAND	2015	55/67	4/15	3/22	14/22
	2023	49/84	6/96	5/45	16/88
NP	2015	448	3186	3803	411
	2023	447	2861	1106	352
PD	2015	0/70	5/04	6/01	0/65
	2023	0/75	4/52	1/75	0/55
LPI	2015	49/81	0/07	0/03	4/29
	2023	43/52	0/22	0/44	10/78
TE	2015	2262990	1718220	1592100	809730
	2023	2434247	2373999	1684822	813818
ED	2015	35/81	27/19	25/19	12/81
	2023	38/52	37/56	26/66	12/87
LSI	2015	31.98	83/92	88/95	22/42
	2023	35/90	89/45	73/26	20/96

Percentage of landscape (PLAND),Number of patches (NP), Patch density (PD), Largest patch index (LPI, Total Edge (TE), Edge Density (ED), Landscape shape index (LSI).

Landscape metrics analysis between 2015 and 2023 reveals significant spatial changes in Tehran’s urban landscape. The PLAND index for cold spots (green spaces) decreased from 55.67% to 49.84% at the 99% confidence level, indicating a reduction in their extent and, consequently, their cooling effect. In contrast, the proportion of hot spots (impervious surfaces such as asphalt and bare land) increased, reflecting urban expansion.

The NP index shows that the number of cold patches remained nearly constant (448 in 2015 vs. 447 in 2023) at the 99% level but declined at 95%, suggesting fragmentation rather than expansion of green areas. Meanwhile, the number of hot patches decreased at both significance levels.

The PD index for cold spots slightly increased (from 0.70 to 0.75), indicating minimal change in spatial dispersion, with green spaces remaining concentrated rather than evenly distributed. The LPI index shows a reduction in the area of the largest cold patches, signifying a decline in the size and quality of urban green spaces. Conversely, large hot patches significantly expanded at both confidence levels.

Both ED and TE values for cold and hot spots increased in 2023, indicating higher landscape fragmentation and more irregular patch shapes. For cold spots, this suggests disintegration into smaller, less effective cooling units. While increased edge length can enhance heat exchange, fragmentation reduces their overall cooling efficiency. The LSI for green spaces also increased, reflecting greater spatial complexity and reduced cohesion.

Overall, these metrics indicate a clear trend toward reduced green space, increased artificial surfaces, and a more fragmented urban landscape—likely driven by urban development, construction, and land-use changes.

Table 6 shows the results of examining the metrics at landscape levels.

**Table 6:** Class-level landscape metrics values.

Metrics	2015	2023
NP	10877	9917
PD	17/21	15/69
LPI	49/81	43/52
TE	5171940	56764869
ED	81/86	89/83
LSI	53/85	58/74
CONTAG	53/06	50/32
SHDI	1/42	1/52

NP decreased from 10,877 in 2015–9,917 in 2023, along with a decline in PD. LPI was also higher in 2015, indicating a more dominant largest patch and greater cohesion. A reduction in LPI suggests a loss of land cover integrity. Despite some metric increases, fragmentation of green spaces has intensified, resulting in higher SHDI and lower CONTAG.

SHDI showed an increasing trend, reflecting growing heterogeneity, while CONTAG declined, and indicating increased fragmentation and reduced connectivity. CONTAG is inversely related to edge density and reflects the degree of landscape degradation.

LSI also increased, suggesting more complex and irregular patch shapes. Higher LSI values point to greater spatial complexity and disordered landscape structure in both cold and hot patches.

## 4. Discussion

### 4.1. Analysis of the spatial distribution of UHIs

This study presents a novel methodological integration of spatial statistics, landscape metrics, and circuit theory to examine how UGS connectivity moderates UHI effects in Tehran. An eight-year comparative analysis (2015–2023) highlights the strategic importance of spatial configurations and thermal linkages in guiding UGS planning for thermal regulation. The analysis reveals that variations in the structural coherence and continuity of green patches closely correlate with changes in LST. Hot and cold thermal clusters, identified at 95% and 99% confidence levels, provide empirical evidence of spatial thermal disparities driven by both biophysical and anthropogenic factors. These clusters expose urbanization-driven inequities in ecosystem-based cooling services, with implications for thermal justice in rapidly growing cities. A marked thermal asymmetry along Tehran’s north–south axis was observed: the north, with denser and more interconnected green areas, maintains resilient cold patches, while the south and west show an increase in hot zones (1,618 ha) and a loss of cold zones (1,400 ha). This trend aligns with earlier findings [25,36] that associate thermal vulnerability with fragmented green space distribution, elevation gradients, and urban morphological patterns. Although cold clusters in the 90–95% confidence range are spreading toward central areas, the most resilient ones (99%) remain in the north. While these core zones have shrunk, their internal connectivity has strengthened, suggesting that spatial cohesion within green networks can partially offset area loss in preserving thermal functionality. A high Kappa index (~0.84) confirms strong spatial consistency of cold patches over time. The growing dispersion of UHIs, especially in western and northwestern areas, reflects the complex transformation of Tehran’s thermal landscape, shaped not only by urban expansion but also by the thermal inertia of non-vegetated surfaces. As Bokaie et al. (2016) note, Tehran’s polycentric, topographically diverse structure heightens UHI vulnerability due to limited UGSs, high population density, emissions, and impervious surfaces [9]. The study finds that peripheral UHIs are more influenced by land cover than localized heat sources. Overall, vegetation structure, density, and spatial distribution are critical in regulating urban thermal regimes. The interaction between green space connectivity and configuration determines the scale and equity of cooling services, offering both theoretical insight and practical guidance for climate-resilient urban planning.

### 4.2. Landscape patterns of thermal islands (or hot and cold patches)

Urban thermal heterogeneity results from the spatial interplay between impervious (hot) and vegetated (cold) surfaces, with the configuration and connectivity of these land cover types serving as critical determinants of local thermal dynamics. While prior studies have highlighted the mitigating effects of UGSs on the UHI effect, limited focus has been placed on the evolving structural relationships between thermally contrasting patches [64–67]. This study addresses that gap by identifying statistically significant (≥95%) hot and cold clusters and analyzing their structural changes through nine landscape metrics, offering a refined, patch-level perspective on LST regulation and the role of functional landscape connectivity in urban thermal governance. Findings align with earlier research [25,27,68], confirming strong associations between LST patterns and landscape metrics such as PLAND, SHAPE_AM, PD, and ENN_AM. Parvar et al. (2025) in Bojnourd, Iran, found that the spatial configuration and connectivity of urban green patches, assessed using metrics such as PD and proportion of like adjacency (PLADJ), strongly influence local cooling, whereas fragmented green areas reduce their thermal regulation capacity [22]. Beyond these static correlations, temporal class-level analysis (2015–2023) reveals a directional transformation: cold patches have become increasingly fragmented, spatially dispersed, and isolated, while hot patches have expanded in size, structural dominance, and cohesion. This spatial polarization reflects not only urban expansion but a broader degradation of ecological and thermal infrastructure. The transition from a connected, resilient green network to fragmented, ecologically diminished units underscores the importance of spatial arrangement and connectivity over mere area. Fragmentation of cold zones undermines their moderating function, while consolidation of hot zones exacerbates localized heat stress and weakens urban adaptive capacity. These results echo Gallay et al. (2023), who identified size, structure, and tree cover as key factors in urban cooling. Spatial connectivity emerges as equally vital for maintaining the ecological functionality of green spaces [15]. Fragmentation of cold patches signals a weakening ecological network, with implications extending beyond thermal regulation to biodiversity loss and reduced ecosystem service provision. To counter these shifts, urban planning should emphasize the preservation and restoration of spatially cohesive green infrastructures. UGSs must be treated as interconnected networks capable of supporting thermal regulation, ecological continuity, and urban resilience. Effective UHI mitigation depends on a dual approach—conserving green space area while reinforcing its spatial coherence—to sustain functional urban landscapes amid rapid urbanization and climate change.

### 4.3. The cooling effect of GCCs connectivity

The integration of cost maps and circuit theory offered an effective means to assess vegetation changes and cold core connectivity in Tehran (2015–2023), revealing key structural shifts in green networks and providing valuable insights for urban climate adaptation.

The pronounced rise in connectivity cost between 2015 and 2023—indicative of deteriorating structural cohesion and reduced thermoregulatory potential of GCCs reflects a broader urban ecological shift. Notably, this trajectory aligns with the longitudinal observations of Wang et al. (2025), who emphasized a progressive weakening in the capacity of urban vegetation to mitigate land surface temperatures. Such convergence underscores an emerging pattern in rapidly urbanizing contexts, where green infrastructure, despite its presence, becomes increasingly fragmented and less effective in delivering climate-regulating ecosystem services [69].

Building on evidences that denser vegetation and spatially balanced green infrastructures enhance urban cooling [70], the present analysis shifts the focus from static spatial metrics to the dynamic functional connectivity of GCCs. While earlier studies have addressed patch-level attributes such as size and shape [71], the role of structural cohesion within the broader green network has received less attention. The results indicate that, despite relatively stable compositional characteristics, connectivity has become increasingly concentrated in the more clustered green zones of northern Tehran, contributing to spatial disparities in thermal performance. Conversely, the southern sectors, characterized by fragmented patches and greater interspace distances, demonstrate weakened interpatch flow and diminished cooling capacity—likely consequences of intensified urban densification and land surface sealing between 2015 and 2023. This pattern of functional disconnect produces a bottleneck effect, restricting cooling flows to limited corridors. Addressing these spatial inefficiencies by identifying and strengthening critical nodes within the network could enhance systemic resilience and mitigate the UHI effect.

Parallel observations in other urban contexts reinforce these findings. In Harare-Zimbabwe, clustered vegetation was shown to deliver stronger cooling effects than fragmented layouts [72], while studies in Karaj-Iran confirmed that disrupted connectivity between thermal zones amplifies UHI intensity.

Mokhtari et al. (2022) reported evidence from Karaj city, where the authors integrated patch-based and network-based analyses to evaluate the spatial pattern of green heat sinks. Importantly, they emphasized that enhancing the connectedness of vegetation cover, particularly in priority locations, is essential for improving the thermal environment. These observations reinforce the present study’s results in Tehran, where the loss of structural cohesion and weakened connectivity of green patches has diminished their thermoregulatory capacity, especially in southern districts.

The moderating role of GCCs as dynamic regulators of urban temperature has similarly been emphasized [40]. Beyond the urban context, Morovati et al. (2020) demonstrated the critical role of habitat connectivity under climate change scenarios in southern and southeastern Iran, where maintaining interpatch linkages was essential for the survival of the endangered Asian black bear. Their findings highlight that connectivity—whether in ecological landscapes or urban green infrastructures—functions as a resilience mechanism, sustaining both biodiversity and climatic regulation.

Collectively, this growing body of evidence highlights the strategic value of spatially coherent and well-connected green infrastructures in mitigating heat stress. From an applied standpoint, the identification of thermal bottlenecks and enhancement of key vegetative corridors emerges as a pivotal strategy for climate-adaptive urban design. These insights provide a robust spatial foundation for guiding policy interventions aimed at restoring ecological flow and reinforcing urban thermal resilience.

From a practical perspective, the identification of key GCC nodes and connectivity bottlenecks provides actionable guidance for urban planners and policymakers in Tehran and other Iranian cities. By prioritizing interventions in areas where green corridors are fragmented or disconnected, authorities can strategically enhance cooling efficiency, mitigate UHI effects, and promote climate-resilient urban design. This approach not only informs spatial planning of new green spaces but also supports restoration and management of existing vegetation, thereby maximizing ecosystem service delivery in the context of rapid urbanization and climate change adaptation.

### 4.4. Limitations and suggestions

One key limitation of this study is the exclusion of smaller green patches due to the 100-meter resolution of Landsat’s thermal band, which limits the detection of fine-scale thermal variations and potentially overlooks the cooling contributions of minor vegetated areas. To overcome this, future research should integrate higher-resolution datasets and advanced spatial analysis techniques to better capture the full spectrum of green space connectivity. Scenario-based modeling of green space expansion and interlinking could provide deeper insights into their effectiveness in mitigating UHI intensity. Additionally, employing machine learning and land-use optimization frameworks may enhance the accuracy of future projections and support more targeted, sustainable urban planning strategies aimed at maximizing the climate-regulating functions of urban greenery.

## 5. Conclusion

This study presents an innovative integration of spatial statistics, landscape metrics, and electrical circuit theory to assess and optimize the connectivity of UGSs for mitigating UHI effects in Tehran. By combining LST hotspot analysis with connectivity modeling of cold green patches across 2015 and 2023, the approach establishes a spatially explicit and practical framework for strategic green space planning, highlighting how UGS configuration directly influences UHI intensity and urban climate resilience. The findings reveal that improving the spatial connectivity of green patches can significantly enhance residents’ access to cooling services, reduce environmental inequality, and support the development of a cohesive green network. Moreover, the results demonstrate that well-connected and optimally distributed green areas help lower LSTs and moderate urban thermal extremes, with southern and western districts requiring greater attention due to their limited green cover. Strengthening the spatial integration of vegetated areas is therefore essential for increasing urban resilience to rising temperatures, and leveraging spatial data with scenario-based modeling can provide more effective strategies for sustainable green infrastructure development. Overall, this study shows that unbalanced green space distribution and the expansion of impervious surfaces have weakened the cooling function of urban vegetation, particularly outside the northern districts, and emphasizes that policymakers must shift from isolated greening projects toward an interconnected green system to reduce UHI impacts and promote a more sustainable, climate-resilient future.
